# Factors Associated with Overweight and Obesity among Kuwaiti Elementary Male School Children Aged 6–10 Years

**DOI:** 10.1155/2010/459261

**Published:** 2010-09-22

**Authors:** Abdulwahab Naser Al-Isa, Jennifer Campbell, Ediriweera Desapriya

**Affiliations:** ^1^Department of Community Medicine and Behavioral Sciences, Faculty of Medicine, University of Kuwait, 24923 Safat 13110, Kuwait; ^2^School of Population and Public Health, University of British Columbia, Canada V6T 1Z3; ^3^Department of Pediatrics, Faculty of Medicine, University of British Columbia, and Centre for Community Child Health Research, Vancouver, BC, Canada V6H 3V4

## Abstract

*Background*. Childhood obesity is becoming a global epidemic which may result in increased morbidity and mortality during young adulthood. *Objectives*. To identify factors associated with overweight and that of obesity among Kuwaiti elementary male school children aged 6–10 years. *Methods*. Weights and heights of 662 students at a randomly selected school were collected to obtain body mass index (BMI). *Results*. The prevalence of overweight and obesity among the students were 20.2% and 16.8%, respectively. There were a variety of factors associated with overweight and obesity; however, having one or more obese brother, an unemployed father, or a high (>11) number of persons living at home was significantly associated with higher risk of overweight and obesity. Increased age and school level as well as having a chronic disease were associated with the risk of overweight. *Conclusion*. Health education programs for families should be implemented to help control overweight and obesity in Kuwaiti children.

## 1. Introduction

Obesity is a worldwide problem occurring as a result of energy intake far exceeding energy expenditure. Lack of physical activity as well as additional environmental and genetic factors also plays a role in the development of obesity. In general, the increasing incidence of obesity globally may be a consequence of social, economic, and cultural problems [1]. Of particular concern is the rise of obesity among children as it results in increased morbidity and mortality during young adulthood and is a powerful predictor of adult obesity [2].

In Kuwait, studies show that obesity is prevalent and levels may continue to increase. Kuwait has one of the highest levels of obesity in the world. [3–5] An estimated one-third of Kuwaiti adults are obese- and there have been several reports describing the pervasiveness of childhood and youth obesity in varying age groups. [3] Rapid modernization leading to changed dietary and physical activity patterns is often hypothesized to be the primary catalyst in Kuwait's obesity epidemic [2, 4, 5]. It is essential to learn more about prevalence, predictors, associated risk factors, and prevention of childhood/adolescent obesity in Kuwait. 

Prevention is often a better solution compared to ineffective treatments; thus, identifying predictors and factors associated with childhood overweight and obesity is an important undertaking. Unfortunately, parents do not always recognize overweight or obesity in their children, or if they do, they tend to believe it to be permanent and unchangeable [6, 7]. In recent years, birth weight has been labeled an important predictor of childhood obesity [8]. In addition, single parenthood has been related to childhood obesity in a number of studies. This may be related to psychosocial factors that may increase a child's risk of obesity such as family dysfunction, maternal psychiatric disorders, and parental neglect [9]. The onset of obesity can be triggered by a variety of factors including issues related to family relationships and has a tendency to occur during growth spurts [10].

To date, few studies have examined the risk factors associated with childhood obesity in Kuwait. Risk factor determinant studies such as ours are important as a first step in designing interventions that meet the unique needs of understudied groups. The purpose of this study was to explore the factors that are associated with overweight and obesity among Kuwaiti elementary male school children aged 6–10 years.

## 2. Methods

### 2.1. Sample

The sample comprised of 662 elementary school students aged 6–10 years drawn from one school in the capital of Kuwait.

### 2.2. Measurements

The three primary measurements taken for this study were age, weight, and height. Age was ascertained to the nearest month from the student's civil identification card which was obtained from school files containing the student's date of birth.

Weight was measured by the lead author, with the student standing and wearing light clothes to the nearest 0.1 kg, using a precalibrated digital SECA scale which was recalibrated between measurements. Height was measured while the subject was standing without shoes to the nearest 0.1 cm, using a specially designed portable stadiometer with a spirit level to ensure that it was parallel to the flat hard floor during measurement. The BMI, which is the weight in kilograms divided by the height in meters squared (kg/m2), was used as the index of adiposity. The BMI was calculated for each age, and the values were compared with the International cutoff points as described below:

“International cutoff points for body mass index for overweight and obesity among male children between 6–10 years, defined to pass through body mass index of 25 and 30 kg/m2 at age 18, obtained by averaging data from Brazil, Great Britain, Hong Kong, Netherlands, Singapore, and United States” (Cole et al., 2000 [11]). (See Table 1)

### 2.3. Data Analysis

The Statistical Programme for the Social Sciences (SPSS) version 17, PC Windows was used for data analysis. In addition to descriptive statistics, the *χ* ^2^ test was used to assess the association between categorical variables. Logistic regression analysis was carried out using a binary variable: nonobese (BMI <25 kg/m^2^) or overweight [(BMI >25 kg/m^2^)/obese (BMI >30 kg/m^2^)] as a dependent variable and a number of other variables as independent variables. The merit of the logistic regression approach is that it leads to the adjusted odd ratios (estimated relative risk or RR) of the independent variables in relation to a reference group. A *P*-value of ≤ .05 was the criterion of statistical significance.

### 2.4. Associated Factors

Age was divided into four categories 6, 7, 8, 9, and 10. The following domains were broken into sub factors.

#### 2.4.1. Dieting and Nutrition

Number of major meals was divided into three: 1,2, and 3; eating between meals into three: yes, no, and sometimes; dieting into three: yes, no, was; number of times dieted into four: none, low (1–2), medium (3–4), and high (≥5); dieting practice into five: on my own, through a consultant, relatives and friends, through the mass media, and does not apply; own nutritional knowledge into three: weak, good, excellent; needing special diet to lose weight into three: yes, no, and do not know.

#### 2.4.2. Activities and Interests

Practice sports (months per year) were divided into three: 1(< month), 2(1–3 months), 3(>9 months); practice sports (hours per week) into four: high (≥5), medium (3–4), low (≤2), and none; exercise into two: yes, no; countries preferred for visiting into four: western, eastern, both, and neither.

#### 2.4.3. Socioeconomics

Type of housing was divided into three: rent, government, and private; number of rooms in the house into three: low (1–2), medium (3–4) and high (≥5); number of persons living at home into four: none (0), low (1–2), medium (3–5), and high (≥6); number of servants at home into four: none (0), low (1–2), medium (3–5), and high (≥6); family income per month into three: low (<$ 1500), medium ($ 1500–$ 3000), and high (>$ 3000).

#### 2.4.4. Family

Number of obese brothers was divided into four: none (0), low (1–2), medium (3–4), and high (≥5); obesity among first-degree relatives into four: none (0), low (1–2), medium (3–4), and high (≥5); obesity among parents into four: neither, father, mother, both; relation between parents into three: first cousin, there is, there is not; father's education into three: low (illiterate or elementary), medium(intermediate or secondary), and high (college or higher); father's employment status into two: working, not working; number of brothers, number of sisters and total number of siblings into four: none (0), low (1–3), medium (4–7), and high (≥8); birth order into first, middle, and last.

#### 2.4.5. Academics

Current grade point average (GPA) was divided into three: high (excellent and very good), medium (good), low (low pass and failure); level of education into four: first, second, third, and fourth year; high school study into two: science, nonscience; highest desired degree into three: high school, college, and higher. 

#### 2.4.6. Health

Dental status was divided into three healthy, treated by dentist, and unhealthy; suffering from a chronic disease into two: yes, no; last dental or physical check-up into four: do not remember, more than two years, a year ago, and a month ago; describe your health into three: bad, good, excellent; feeling tired often into two: yes, no.

## 3. Results

The prevalence of overweight (BMI >25–30 kg/m^2^) and obesity (BMI >30 kg/m^2^) among the students was 20.2 and 16.8%, respectively.

Table 2 shows the factors associated with overweight (BMI >25–30 kg/m²) and obesity (BMI >30 Kg/m²) among Kuwaiti elementary male school children aged 6–10 years from chi-squared analysis. These factors were age (*P *< .05), dental status (*P *< .01), chronic disease (*P *< .05), number of obese brothers (*P *< .001) and sisters (*P *< .01), father's occupation (*P *< .01), number of persons living at home (*P *< .01), desiring a higher degree (*P *< .05), dieting (*P *< .05), school level (*P *< .01), number of times dieted (*P *< .001), and needing special nutritional program (*P *< .001).

Table 3 shows the risk factors associated with overweight (BMI >25–30 kg/m²) and obesity (BMI >30 kg/m²). The risk of being overweight was significantly higher among those at age eight (*P *< .05, OR = 1.9) and ten (*P *< .01, OR = 2.1) in comparison with the reference group of six years of age, decreased significantly among those with treated teeth (*P *< .05, OR = 0.6) in comparison with the reference group with healthy teeth, increased significantly among those with chronic disease (*P *< .01, OR = 1.8) in comparison with the reference group who had none, increased significantly among those with low (1) numbers (*P *< .01, OR = 1.9) and high (≥3) numbers (*P *< .05, OR = 2.6) of obese brothers in comparison with the reference group who had none, increased significantly when father was not working (*P *< .01, OR = 2.0) in comparison with the reference group with a working father, increased significantly with a high (≥11) number (*P *< .01, OR = 2.6) of persons living at home in comparison with the reference group with a low (1–6) number, decreased among those who did not diet (*P *< .01, OR = 0.4) in comparison with the reference group who did, increased significantly among the fifth (*P *< .001, OR = 2.8) level in comparison with the reference group of the first level, decreased significantly among those who dieted once (*P *< .05, OR = 0.3) in comparison with the reference group who did not need to diet (*P *< .01, OR = 0.5), and also decreased among those who did not know they needed special diet program in comparison with the reference group who did.

The risk of obesity (BMI >30 kg/m²) decreased significantly among those with treated teeth (*P *< .001, OR = 0.4) in comparison with the reference group with healthy teeth, increased significantly among those with low(1) number (*P *< .001, OR = 2.6), medium(2) number (*P *< .05, OR = 2.6), and high (≥3) number (*P *< .05, OR = 3.2) of obese brothers in comparison with the reference group who had none, increased significantly among those with nonworking father (*P *< .01, OR = 2.3) in comparison with the reference group with a working father, increased significantly in homes with a high (≥11) number of people living in them (*P *< .05, OR = 2.1) in comparison with the reference group with low (1–6) number, decreased significantly among those who did not diet (*P *< .05, OR = 0.5) in comparison with the reference group who did, decreased significantly among those who dieted once (*P *< .05, OR = 0.3) in comparison with the reference group who never dieted, and decreased significantly among those who did not need a special diet program (*P *< .001, OR = 0.4) as well as among those who did not know they needed special diet program (*P *< .01, OR = 0.4) in comparison with the reference group who needed it.

## 4. Discussion

Factors that have been found to be associated with obesity in other studies include parental education and smoking [12], socioeconomic status [13], increased sedentary behavior which is higher in girls than boys [14], watching television more than two hours daily, the likelihood of having one or more serving per day of high-energy drinks and one or more serving per day of high-energy snacks, and being less likely to have two or more serving per day of fruits or to participate in any organized physical activity [14]. Others have found such factors as general diet, rapid eating, short sleep duration, early bed time, long periods of television viewing, frequent bowel movement, and avoidance of physical activity to be associated with obesity [15]. In addition, maternal smoking during pregnancy might be a risk factor for childhood obesity [16].

Fourteen factors that were significantly associated with overweight and obesity were found in this study. The factors that were not found in the reviewed literature were dental status, suffering from a chronic disease, number of obese brothers, father's employment status, number of persons living at home, highest desired degree, school level of study, number of times dieted, and needing a special dietary program to lose weight. When the significantly associated factors were subjected to logistic regression, the risks for overweight and obesity were having one or more obese brother, an unemployed father, or a high (>11) number of persons living at home. Increased age and school level as well as having a chronic disease was associated with the risk of overweight only. 

It is possible that this study found additional factors associated with overweight and obesity because perhaps more factors were studied than done previously. The significantly associated risks should be dealt with through appropriate health education interventions, especially targeting those children in homes where the risks exist. Given the magnitude of this problem, a concerted effort by parents, caregivers, school administration, and the government will be needed to reduce childhood obesity-associated risk factors and related expenditures from rising in the foreseeable future. 

Parental involvement is a key to the success of obesity-prevention programs at a young age as parents and caregivers have primary control over their children's' nutrition and activity levels. Accordingly, parental obesity is the best predictor of childhood obesity. Parents should be encouraged to teach and role model healthy lifestyle behaviors for their young children. Pediatricians and other health care professionals should also be involved in obesity prevention, as they are ideally placed to identify young children at risk for obesity [17, 18].

It is clear that a multilevel and comprehensive approach is needed to combat overweight and obesity in Kuwait. Programs such as the Let's Move campaign in the USA [19], school-based physical activity programs in Switzerland [20], and the programs described in a publication by EuroHealthNet [21] should be used as models. In order to be effective, these prevention programs must resonate with unique Arabic culture and cultural values [22]. Reducing overweight and obesity will lead to increased health equity in Kuwait.

## Figures and Tables

**Table 1:** 

Age (years)	Body mass index 25 kg/m2	Body mass index 30 kg/m2
6	17.6	19.8
7	17.9	20.6
8	18.4	21.6
9	19.1	22.8
10	19.8	24.0

**Table 2:** Factors associated with overweight (BMI >25–30 kg/m^2^) and obesity (BMI >30 kg/m^2^) among Kuwaiti elementary male school children aged 6–10 years (*n* = 662)—Results of chi-squared analysis.

Factor	Nonobese		Overweight		Obese		*P*-value
	*n*	(%)	*n*	(%)	*n*	(%)	
*Age group (years)*							< .05
6	74	(17.7)	14	(10.4)	14	(12.6)	
7	97	(23.3)	22	(16.4)	19	(17.1)	
8	80	(19.2)	27	(20.1)	31	(27.9)	
9	91	(21.8)	29	(21.6)	30	(27.0)	
10	75	(18.0)	42	(31.3)	17	(15.3)	

*Dental Status*							< .01
Healthy	145	(34.8)	48	(35.8)	58	(52.3)	
Treated	198	(47.5)	61	(45.5)	32	(28.8)	
Not healthy	74	(17.7)	25	(18.7)	21	(18.9)	

*Chronic disease*							< .05
No	368	(88.2)	108	(80.6)	90	(81.1)	
Yes	49	(11.8)	26	(19.4)	21	(18.9)	

*No. obese brothers*							< .001
None	347	(83.2)	105	(78.4)	9	(2.2)	
Low (1)	35	(8.4)	11	(8.2)	23	(20.7)	
Medium (2)	16	(3.8)	6	(4.5)	7	(6.3)	
High (>3)	9	(2.2)	5	(3.7)	21	(3.2)	

*Father's occupation*							< .01
Working	386	(92.6)	119	(88.8)	92	(82.9)	
Not-working	31	(7.4)	15	(11.2)	19	(17.1)	

*No. living at home*							< .01
Low (1-6)	245	(58.8)	65	(48.5)	56	(50.5)	
Medium (7-10)	153	(36.7)	57	(42.5)	43	(38.7)	
High (>11)	19	(4.6)	12	(9.0)	12	(10.8)	

*Highest degree*							< .05
Low	44	(10.6)	9	(6.7)	11	(9.9)	
Medium	278	(66.7)	87	(64.9)	60	(54.1)	
High	95	(22.8)	38	(28.4)	40	(36.0)	

*Dieting*							< .05
Yes	16	(3.8)	11	(8.2)	11	(9.9)	
No	380	(91.1)	119	(88.8)	92	(82.9)	
I was	21	(5.0)	4	(3.0)	8	(7.2)	

*School level*							< .01
1	87	(20.9)	15	(11.2)	18	(16.2)	
2	101	(24.2)	25	(18.7)	18	(16.2)	
3	85	(20.4)	25	(18.7)	29	(26.1)	
4	89	(21.3)	28	(20.9)	28	(25.2)	
5	55	(13.2)	41	(30.6)	18	(16.2)	

*No. times dieted*							< .001(†)
None	383	(91.8)	115	985.8)	85	(76.5)	
Low (1)	16	(3.8)	4	(3.0)	7	(6.3)	
Medium (2-3)	12	(2.9)	11	(8.2)	13	(11.7)	
High (>4)	6	(1.4)	4	(3.0)	6	(5.4)	

*Needing special diet program*							< .001
Yes	91	(21.8)	34	(25.4)	50	(45.0)	
No	261	(62.6)	81	(60.4)	48	(43.2)	
I don't know	65	(15.6)	19	(14.2)	13	(11.7)	

*P < .05, **P < .01, ***P < .001, (†) chi-square value may not be reliable as the number of cases are low in certain cells.

**Table 3:** Risk factors associated with overweight (BMI >25–30 kg/m^2^) and obesity (BMI >30 kg/m^2^) among Kuwaiti elementary male school children aged 6–10 years (*n* = 662)—Results of logistic regression.

Factors	Overweight		Obese	
	OR	(95% CI)	OR	(95% CI)
*Age group (years)*				
6 (reference)	1.0		1.0	
7	1.1	(0.6–1.9)	1.0	(0.5–2.1)
8	1.9	(1.1–3.3)*	1.8	(0.9–3.6)
9	1.7	(0.9–2.9)	1.6	(0.8–3.1)
10	2.1	(1.2–3.6)**	0.9	(0.4–1.9)

*Dental Status*				
Healthy (reference)	1.0		1.0	
Treated	0.6	(0.5–0.9)*	0.4	(0.3–0.7)***
Not healthy	0.8	(0.5–1.3)	0.7	(0.4–1.2)

*Chronic disease*				
No (reference)	1.0		1.0	
Yes	1.8	(1.2–2.8)**	1.5	(0.9–2.5)

*No.* *obese brothers*				
None (reference)	1.0		1.0	
Low (1)	1.9	(1.2–2.9)**	2.6	(1.5–4.3)***
Medium (2)	1.9	(0.9–3.8)	2.6	(1.2–5.9)*
High (>3)	2.6	(1.1–6.3)*	3.2	(1.3–8.2)*

*Father's occupation*				
Working (reference)	1.0		1.0	
Not–working	2.0	(1.2–3.3)**	2.3	(1.3–4.0)**

*No.* *living at home*				
Low (1–6) (reference)	1.0		1.0	
Medium (7–10)	1.3	(0.9–1.8)	1.1	(0.7–1.8)
High (>11)	2.6	(1.3–4.8)**	2.1	(1.0–4.4)*

*Dieting*				
Yes (reference)	1.0		1.0	
No	0.4	(0.2–0.8)**	0.5	(0.2–0.9)*
I was	0.4	(0.2–1.1)	0.8	(0.3–2.3)

School level				
1 (reference)	1.0		1.0	
2	1.1	(0.7–1.9)	0.8	(0.4–1.6)
3	1.6	(1.0–2.8)	1.5	(0.7–2.8)
4	1.7	(1.0–2.8)	1.4	(0.7–2.6)
5	2.8	(1.6–4.8)***	1.1	(0.5–2.2)

*No.* *times dieted*				
None (reference)	1.0		1.0	
Low (1)	0.3	(0.1–0.8)*	0.3	(0.1–0.8)*
Medium (2–3)	0.4	(0.1–1.4)	0.6	(0.2–2.2)
High (>4)	1.2	(0.3–4.1)	0.9	(0.3–3.2)

*Needing special diet program*				
Yes (reference)	1.0		1.0	
No	0.5	(0.4–0.8)**	0.4	(0.2–0.5)***
I don't know	0.5	(0.3–0.9)*	0.4	(0.2–0.8)**

*P < .05, **P < .01, ***P < .001, OR (Odds Ratio), CI (Confidence interval).
